# Humanitarian sector (international non-governmental organisations) support to the community in Goma city/DRC during the COVID-19 pandemic period: Expectations and reality

**DOI:** 10.1371/journal.pgph.0002086

**Published:** 2023-10-20

**Authors:** Roger Paluku Hamuli, Susannah H. Mayhew, Mateus Kambale Sahani

**Affiliations:** 1 Department of Research and Diseases Prevention, Centre Medical Hope, Goma city, Democratic Republic of Congo; 2 Faculty of Public Health and Policy, Department of Global Health and Development, London School of Hygiene & Tropical Medicine, London, United Kingdom; PLOS: Public Library of Science, UNITED STATES

## Abstract

COVID-19 was the largest public health emergency to disrupt social life and health systems worldwide. The pandemic affected all world continents creating fear and stress in many aspects of social life. The pandemic spread from China to Europe, then to Africa carrying with it all the negative impacts affecting population wellbeing. The COVID-19 pandemic was declared in the Democratic Republic of Congo (DRC) in March 2020 and created huge shock and stress countrywide. Goma city accommodates more than 30 international non-governmental humanitarian organisations (HO) who have sought to support local communities to help them overcome COVID-19 stress. Few studies to date have considered the role of these HO from the perspective of the beneficiary populations. This is a descriptive, analytical study, reporting data collected from a survey questionnaire to 100 community members (including 21 healthcare professionals) in Karisimbi health zone in Goma city in DRC. The study’s main aim was to explore how community members viewed the contribution and impact of HO actions during COVID-19 in Goma city. We identified some important mis-matches between community expectations and HO actions which must be addressed in future outbreaks. First, community members had big expectations of HO in terms of practice support to tackle the pandemic (including providing handwashing devices and mobile support teams), yet the vast majority of respondents reported seeing little or no such actions. This can create resentment against HO and it is critically important that they rapidly engage with communities at the start of any outbreak to understand their needs and concerns and develop strategies to directly respond to these. Second, HO played a very limited role in dissemination of information about COVID-19 and were not trusted messengers. Our findings showed that most people’s preferred source of information about COVID-19, specifically vaccines, was local healthcare workers–particularly those who were known well and therefore trusted. HO (and national responders) should therefore map trusted spokespersons (including healthcare professionals) in the targeted communities and involve them in the planning and implementation of interventions as essential steps in the response. Among our respondents, social media played a large role in information sharing. Further research is needed to understand the role that social media (particularly Facebook and WhatsApp which were most frequently used) could play in sharing messages from trusted sources, including official government communications. Collectively, these actions could help create a positive attitude towards COVID-19 vaccine and similar interventions in future outbreaks.

## 1. Introduction

### 1.1 General background on COVID-19

COVID-19 was the largest public health emergency to disrupt social life and health systems worldwide [1,2]. The crisis impacted the most vulnerable populations who needed sufficient support to prevent loss of life in their respective communities. The pandemic affected around 200 countries worldwide up to March 2020, increasing the case fatality rate and raising concerns for global health security [1–3].

COVID-19 was predictable in China as an epidemic, but not as a pandemic [4]. The infection was globalised by mutation of spike protein of the SARS-Cov-2 creating new strains combined with the high mobility of international trade and travel opportunities which fueled its spread throughout the world [4]. This has been a lesson to global health leadership and has shown that the strain of the virus that has devastated the world was different from the original virus that caused the epidemic in Wuhan [4].

Healthcare has been seriously affected by the COVID-19 pandemic in most countries [5]. Primary care was disrupted by the abandonment or postponement of routine care in communities, yet this level is critical for compliance with infection control guidelines and measures which can prevent death and overcome the pandemic [3,5]. There is a need to strengthen sustainable connections through all levels of the healthcare system.

Although the COVID-19 pandemic has been most virulent and deadly in European and Asian countries, the African continent was also affected with serious negative impacts on all sectors of social life including population health, economy, business, and routine social life activities [6]. Africa is a continent with populations which are most vulnerable to infectious diseases and it was predicted that they will be more affected than the rest of the world [6,7]. According the Infectious Diseases Vulnerability Index (IDVI), 23 countries among the 25 most vulnerable, are in Africa [6,8] and DRC ranks 11^th^ among these 23 African vulnerable countries [8].

The weaknesses of the health system in a country expose the population to risks and make them vulnerable to health shocks. Even developed countries such as USA, UK, France, and Italy, which have strong health systems, were still struggling to cope with COVID-19 surveillance and control. African countries where health systems show the most weaknesses could not stop the spread of the disease as they do not have the needed equipment and supply for testing and diagnostic [6,7]. Even though COVID-19 was predicted as a devasting disaster in Africa, the situation on ground has shown a paradox [7,9]. The number of confirmed cases and the case fatality rate have been lesser than what was experienced in other continents, although the reasons for this are not entirely clear [7,9,10].

Some of the African countries have had an increase of cases during the second wave with a rate similar to what was observed in western countries. The most highlighted countries that were seriously affected are Libya, Morocco, Cabo Verde, and South Africa [10]. This shows to what extent the region is fragile and needs support to protect its vulnerable populations.

In DRC the pandemic was declared in the country in March 2020 [6,11], and has created fear and stress countrywide affecting social life in diverse communities. It was observed that 18 countries in Africa had reported higher CFR (Case Fatality Rate) which is above the global rate of 2.2% and DRC’s case fatality rate was 3.3% which ranks among the highest in the continent compared to the global rate of 2.2% up to 31 December 2020 [10]. The pandemic was projected to seriously affect the country’s economy and boosting the agricultural sector was proposed as solution to reduce the country’s reliance on food imports and maintain the economy at an acceptable level [11].

While North Kivu province is known for its devastating experience of Ebola, a study has shown that in big cities such as Goma (the Provincial capital), COVID-19 has had much negative impact on livelihoods there than the Ebola epidemic did [12]. It was realised that 85% of the populations of Goma experienced a decrease of their revenue due to COVID-19 while only 14% have reported such impact due to the Ebola epidemic. This is due to the rapid transmission characteristic limiting the interconnection with people for a community in which the life depends the most on business and small urban daily activities [12].

The population of Goma city has experienced multiple, closely sequenced health shocks–or syndemics [13]. The longest epidemic of Ebola (2018–2020) created a heavy burden to the DRC health system, then COVID-19 coming in addition creating confusion among stakeholders not knowing what should be prioritised. Furthermore, the Nyiragongo volcanic eruption on 22 May 2021 was another disaster that came to complicate the situation on ground, increasing the risk of spreading the COVID-19 among displaced people [12,13].

### 1.2 Community engagement

COVID-19 is a pandemic disease for which the surveillance and control processes depend on the containment measures which are driven by trust and compliance by the community members. A study conducted by Hocheal Lee and colleagues [14], has shown that people in DRC trust procedures performed in health facilities regarding diagnosis, testing, and treatment more than people in Ethiopia and Korea did. However, the same study found that the rate of practicing the COVID-19 preventive measures is very low comparing to Ethiopia and Korea. Similarly, a study in Kinshasa showed high levels of belief in the seriousness of COVID-19 but few people adopted positive healthy attitudes to enabling the implementation of preventive measures [15]. A study done in Zimbabwe at the early period of COVID-19 stress occurrence, has also shown that the population did not comply with the restrictive measures put in place by the government due to lack of needed supplies for the normal daily life such as water and food [16].

Moreover, it is important to understand the factors that constitute the barrier to the COVID-19 community response. It was found that people in Kinshasa were getting information on COVID-19 from different sources including social media from friends, radio, television, newspaper, etc. [16]. The same observations were reported in the study conducted in Katanga province/DRC by Kuhangana and colleagues [17]. According to a study conducted in Kinshasa, health professionals and people working in private and governmental organisations are more likely to be adherent to COVID-19 preventive measures than other categories of the population [18].

A study conducted in the community in Goma city has shown that people have positive attitudes towards the containment measures put in place and are ready to use them. However, they need support with good risk communication that involves the full participation of community members and takes into account people’s needs [19]. Community leaders such as faith leaders have expressed the need for collaboration with community members to coproduce effective messages to mitigate COVID-19 stress [20,21]. It is important to empower community leaders to strengthen their capacity in coproducing public health messages that are acceptable in the community and avoid any potential confusion. Most importantly, this may reinforce the dialogue between the health system’s responders and the community members in a particular context of conflict [20,21]. The global humanitarian plan for COVID-19, in objective 3, gives an option of preventing, suppressing, interrupting the transmission of the disease at the community level [22]. This would entail working closely with community members to comply with the containment measures. However, the implementation of the plan was a top-down procedure without involving the community but based on a dictatorship model guided by the politics [23].

### 1.3 Role of humanitarian (international) organisations in COVID-19 response in the DRC

HO are frequent responders to pandemic outbreaks and much has been written about both the lateness and inappropriateness of some of their actions in response to the West Africa and DRC Ebola outbreaks between 2013 and 2020 [24,25]. COVID-19, as worldwide pandemic, again triggered the need for international collaboration. A call for this collaboration was launched by the WHO and led to close cooperation between the Chinese and Japanese governments [26]. China, where COVID-19 originated, was given a donation by 62 countries and 7 international organisations, though little is known about who these were [26].

The COVID-19 response has been shaped in an uncoordinated way and countries have defined their own mechanisms of response dominated by political implications [23]. Politics often took over from science and many response mechanisms were not evidence-based, leading to criticism about whether responses were following WHO guidelines or just dictated by countries’ politicians [23].

Data and case studies on the roles and actions of international organisations in the response to the pandemic, particularly at the community level, are lacking. Our study is a contribution to filling this gap.

### 1.4 Study focus and research questions

This study focuses on community perspectives of the role, contributions, and impact of humanitarian organisations (HO) based in Goma city during the COVID-19 response. Thus, the main aim is to explore how community members viewed the contribution and impact of HO actions during COVID-19 in Karisimbi health zone in Goma city, DRC. Therefore, our research questions are formulated as follows. First, what was the mental health impact among community members when COVID-19 was declared in Goma city? Second, did the community expect humanitarian organisations to support them coping with COVID-19 stress? Third, did the COVID-19 activities of HO meet communities’ expectations?

## 2. Methods

### 2.1 Study location and population

This study was conducted by a research team from Hope Medical Center (HMC), with technical support from researchers from the London School of Hygiene and Tropical Medicine (LSHTM), in Goma city which is the capital of the North Kivu province in the Democratic Republic of Congo. The city is located between Kivu Lake at the south and Nyiragongo volcanic mountain at the North. The city has a population of nearly 2 million people [19] and is divided in 2 health zones: Karisimbi health zone and Goma health zone. Our study was conducted in Karisimbi health zone which is the most populous zone, housing 70% of the city’s population (around 1,400,000 people) with residents who are generally of lower socio-economic status compared to those living in Goma health zone and, therefore, considered the most in need of support.

In this manuscript, we define humanitarian organisations as international non-governmental organisations involved in a humanitarian mission. During COVID-19, most local non-governmental organisations were not able to operate, leaving individuals in communities to help each other, for example by making face masks locally to avoid harassment by policemen.

### 2.2 Study design, data collection, analysis, and sample size

This is a cross-sectional study by survey questionnaire. The data were collected through an administered survey questionnaire in communities in Karisimbi health zone from 01 February to 31 May 2022 by the research team from Hope Medical Centre, almost 2 years after COVID-19 was officially declared in DRC (March 2020). The survey questionnaire was tested before implementation in the non-target health zone (Goma health zone) using volunteer responders. The average time to complete the survey by a participant was estimated to be 10 minutes if the participant did not have questions for clarifications and 15 minutes when clarifications were needed. The questionnaire was in multiple-choice format with an option for open ended answers, specified as “Others (specify)”. This allowed participants to provide details in case their choice was not among the provided options. This was the case for all the respondents. As this is a quantitative study and to give the same chance to all residents to participate, we used systematic random sampling: participants were selected randomly in the households with a defined distance of 20 households between 2 participants. Data were collected in small villages of Karisimbi Health zone of about 3,000 householders and only 1 participant was needed per household. Not all households were taken per village. The first household was taken randomly, then consider the interval of 20 households. After a certain number of participants are enrolled, we could move on to another village. A sample frame of households was made in advance and every 20^th^ was taken during the selection of participants. Only adult residents of 18 years old or more were included in the study. Visitors of less than 3 months in the city at the time of data collection were excluded from the study.

The data were managed and analysed using SPSS software v28.0 of 2021. All the variables were categorical except the participant identification number. Hence, statistics such as mean, standard deviation, median, mode, percentile, are not provided.

Both univariate and bi-variate analyses were used. Univariate analysis was used for dependent variables. The One-Sample p-value test was performed to determine the significance. Moreover, bivariate analysis was performed to compare the dependent variables to other study population’s characteristics such as level of education, sex (female/male), age groups, the social status (non-health professionals or healthcare professionals). The Chi-square(X^2^) test was used for this analysis.

For a good representation of the studied population, the sample size was calculated using g-power software version 3.1 with large effect size of 0.5, the level of significance α = 0.05, and actual power (level of confidence) of 0.95. The calculation was considered for both central and non-central distributions for Chi-square test with two tails and the critical X^2^ of 11.0705. The statistical test category was the “goodness-of-fit tests: contingency tables” and the type of power analysis was “A priori: computer required sample size-given α, power, and effect size. This calculation has led to the minimum sample of 80 participants. However, to increase the power effect of the sample, the researchers have rounded the actual sample size to 100 participants.

### 2.3 Data presentation

In this study, our data are presented in bar charts for univariate analysis and in tables for bivariate analysis.

### 2.4 Ethical considerations and recruitment process

**Fig 1:**
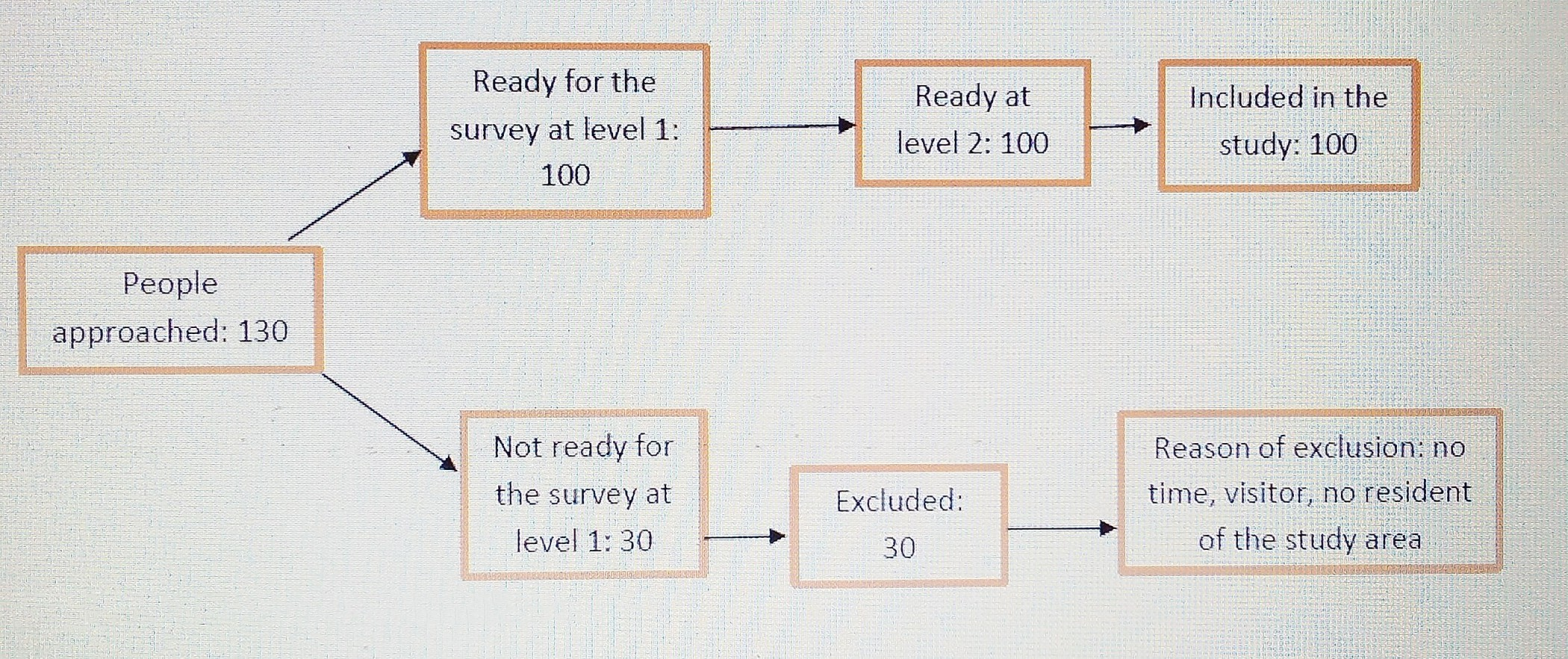
Participants recruitment process.

All the participants included in this study have consented both verbally and in writing. This study was approved by the internal Institutional Review Board (IRB) of Hope Medical Center and has included adult participants only (18 years old or above). All the participants included in the study have consented voluntarily to take part. The consent was verbal and written. Those who accepted to participate were given the survey questionnaire which was administered with guidance from a research team member. The first question of the survey was: do you consent to participate to this study (yes/no). Only those who responded “yes” could continue filling the questionnaire. Those who responded “no” at the second level, were excluded and could not continue the following questions. The total number of people approached was 130, among them 30 were not ready to take the survey at the first level and were excluded. Most of them were excluded for 3 reasons: not having time to take the survey, being recent visitor in the neighborhood, and residing outside the Karisimbi health zone. Fig 1 below summarises the participants’ recruitment process.

### 2.5 Inclusivity in global research

Additional information regarding the ethical, cultural, and scientific considerations specific to inclusivity in global research is included in the Supporting Information (S1 Checklist).

## 3. Results

This section first describes the respondent characteristics and mental health impact of COVID-19, then presents the main findings of the study. These consist of respondents’ perceptions and experiences of HO and their actions; specific findings around COVID-19 vaccines; and an analysis of perceptions by background characteristics (social status and education).

### 3.1. Summary of general characteristics of participants

Our sample consisted of 54% females and 46% males. The majority were less than 41 years of age: 35% were between 18–25 years; 37% between 26–40 years; 20% between 41–55 years; and 8% were older than 55 years. 79% of participants were non-healthcare professionals and 21% were healthcare professionals (resident in the community). In terms of education level, 18% of our participants attained primary school, 51% attained secondary school, and 31% attained university level.

### 3.2 Participants’ fear (mental health impact) of COVID-19

The results in the figure below show how the rise of COVID-19 in Goma city impacted the mental health of the population in terms of the fear they felt about the disease and their daily risks. The word “fear” is a proxy for mental health problems.

**Fig 2:**
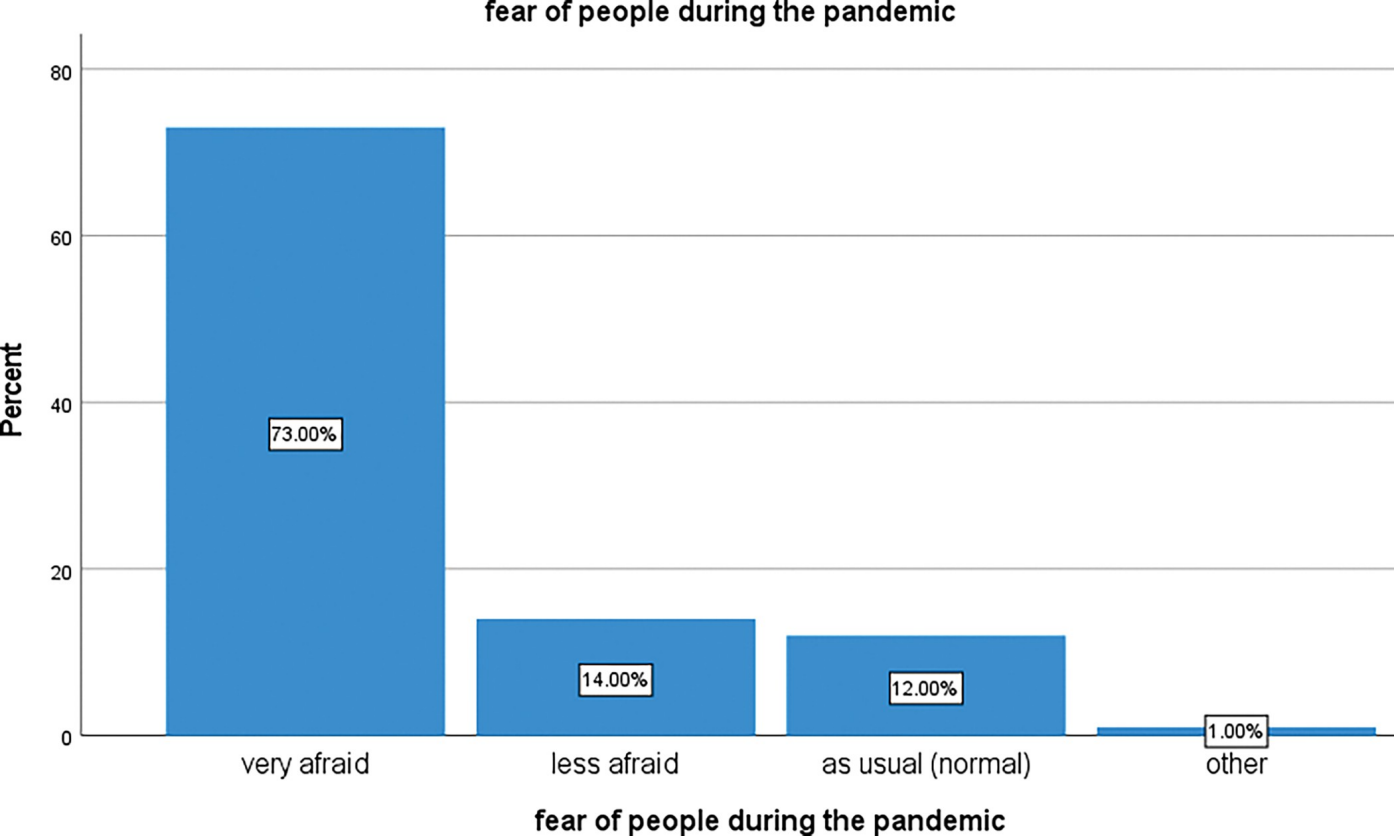
Feeling of participants in presence of COVID-19 pandemic.

During the course of the COVID-19 pandemic, the population in Karisimbi health zone was mentally affected. Among the study participants, 73% were very afraid of the disease, 14% less afraid. These two categories bring the percentage of people who expressed fear to 87% while only 12% of them kept the normal feeling and 1% expressed other feeling such as “not sure, don’t know”. Fig 2 below shows how respondents were mentally affected by COVID-19 pandemic in Goma city (Karisimbi Health Zone).

This shows that the community members of Karisimbi health zone were seriously mentally affected by the presence of COVID-19. The One-sample test shows a statistically significant difference between those who were very afraid and the ones who were less or not (p < 0.0001).

### 3.3 Respondent perceptions and experiences of HO and their actions

We were interested to understand what community members’ perceptions and experiences of the actions of international humanitarian organisations were during the COVID-19 response. This section reports respondents’ views on how helpful they thought humanitarian organisations were to the local response. If they were absent; what community members expected HO to do to support the local COVID-19 response; and what actions they actually saw HO performing in their communities. We present an assessment of the differences between expectations and actions.

#### 3.3.1 Respondent views of the helpfulness of HO in the community

**Fig 3:**
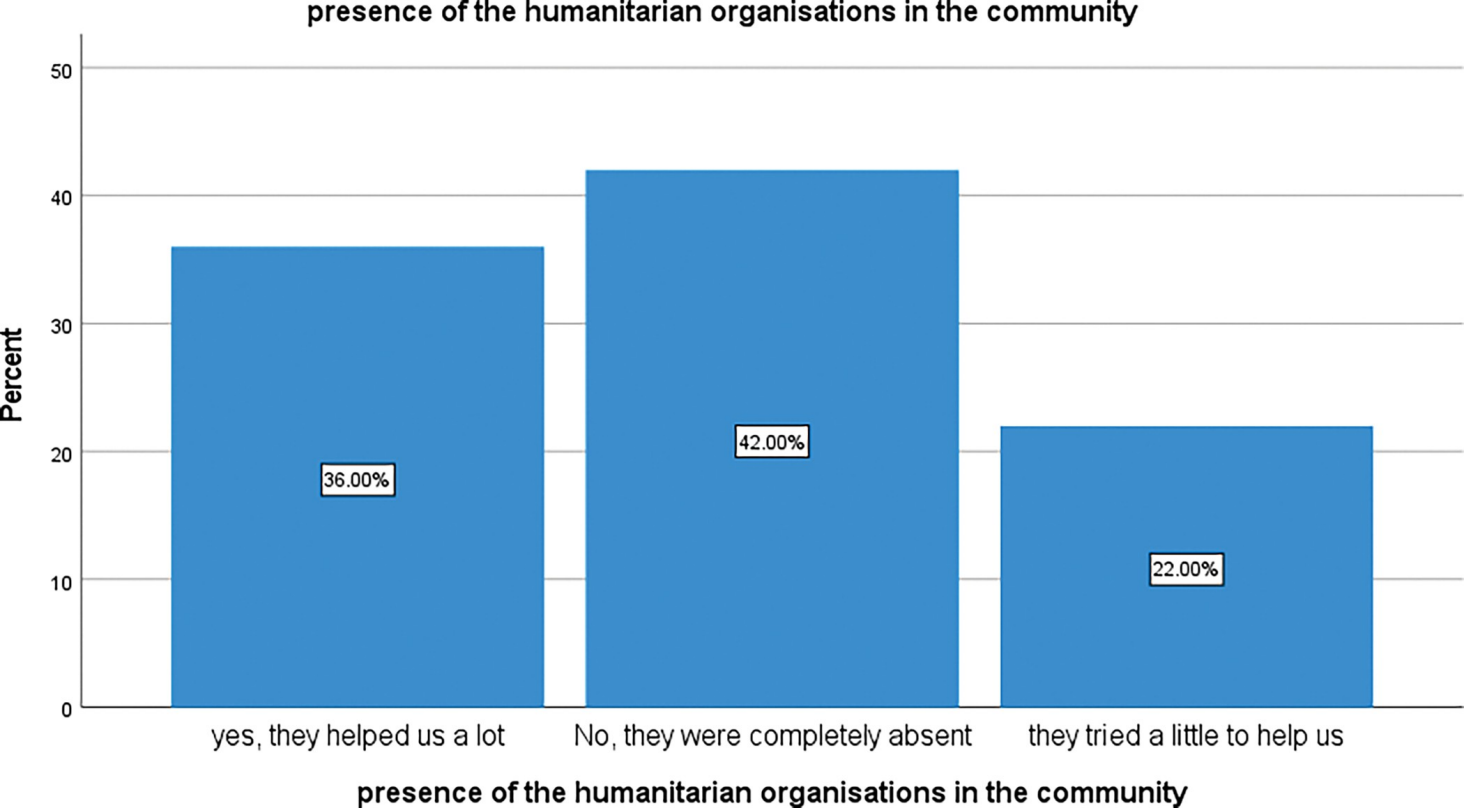
Presence of HO in the community for intervention.

The results here show how the respondents judged the presence of HO in COVID-19 response actions at the community level. Forty-two percent (42%) of respondents reported that they observed a complete absence of HO in COVID-19 mitigation activities in their community whereas 36% felt that HO had done a lot, and 22% judged their interventions as “little”. There is a statistically significant difference between these observations, p = 0.042. These findings are summarised in Fig 3 below.

#### 3.3.2 Respondents expectations of HO

**Fig 4:**
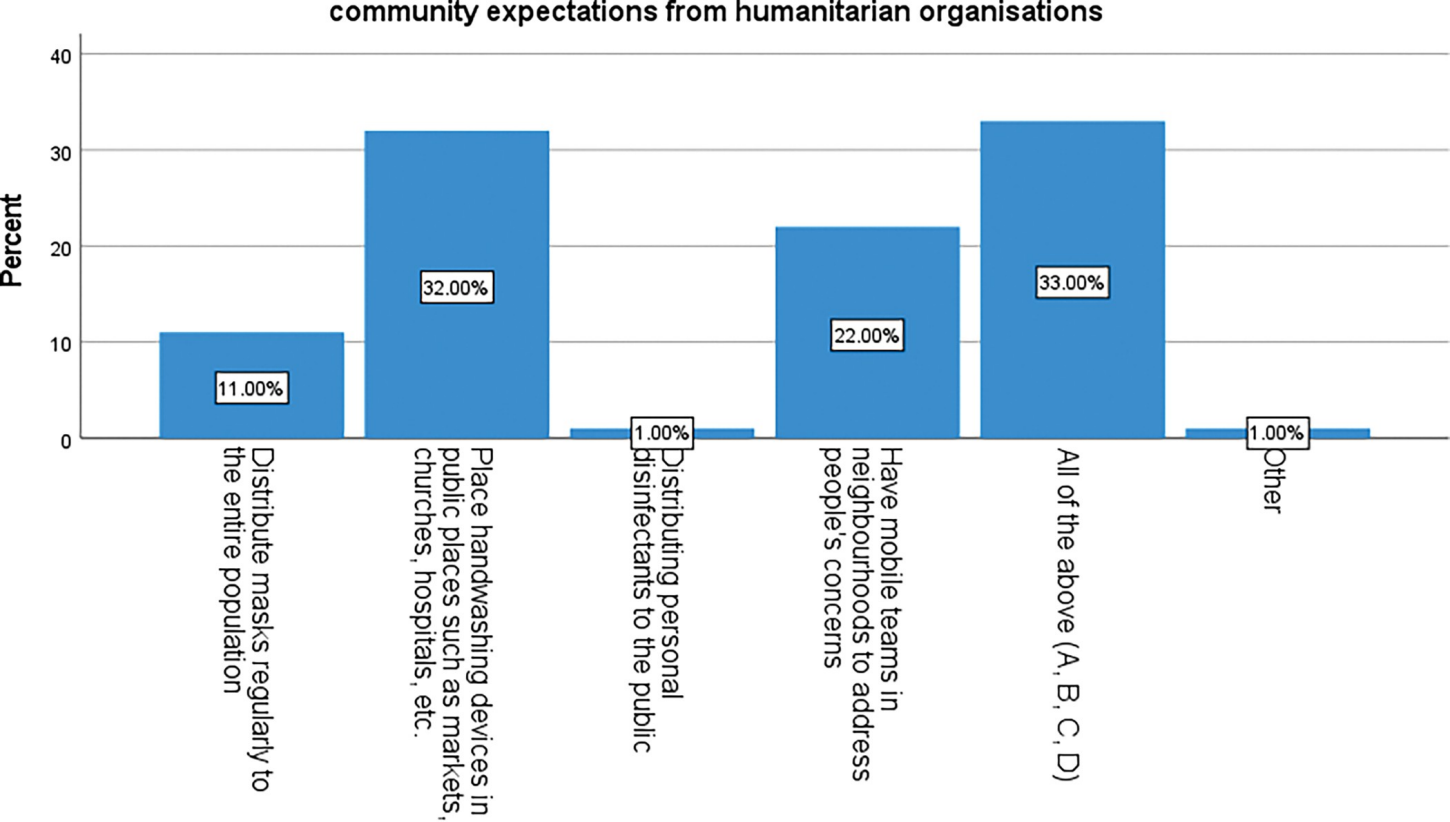
Community expectations from HO in coping with COVID-19 at the grassroot level.

These results show what respondents reported that they expected HO to do to support the local COVID-19 response. We found that 32% expressed an expectation that HO distribute handwashing devices in public places, 22% expected HO to support them by sending their staff to communities with mobile teams in the community neighbourhoods to address local concerns, 11% of participants expected HO to distribute masks to the population, while 1% thought they should distribute personal disinfectant to the population. Fig 4 below shows the findings of our investigations regarding expectations by the community members from HO.

In addition, 33% thought HO should be doing all these actions. The difference observed between these categories is statistically significant, p < 0.001.

#### 3.3.3 Interventions seen on ground by respondents

**Fig 5:**
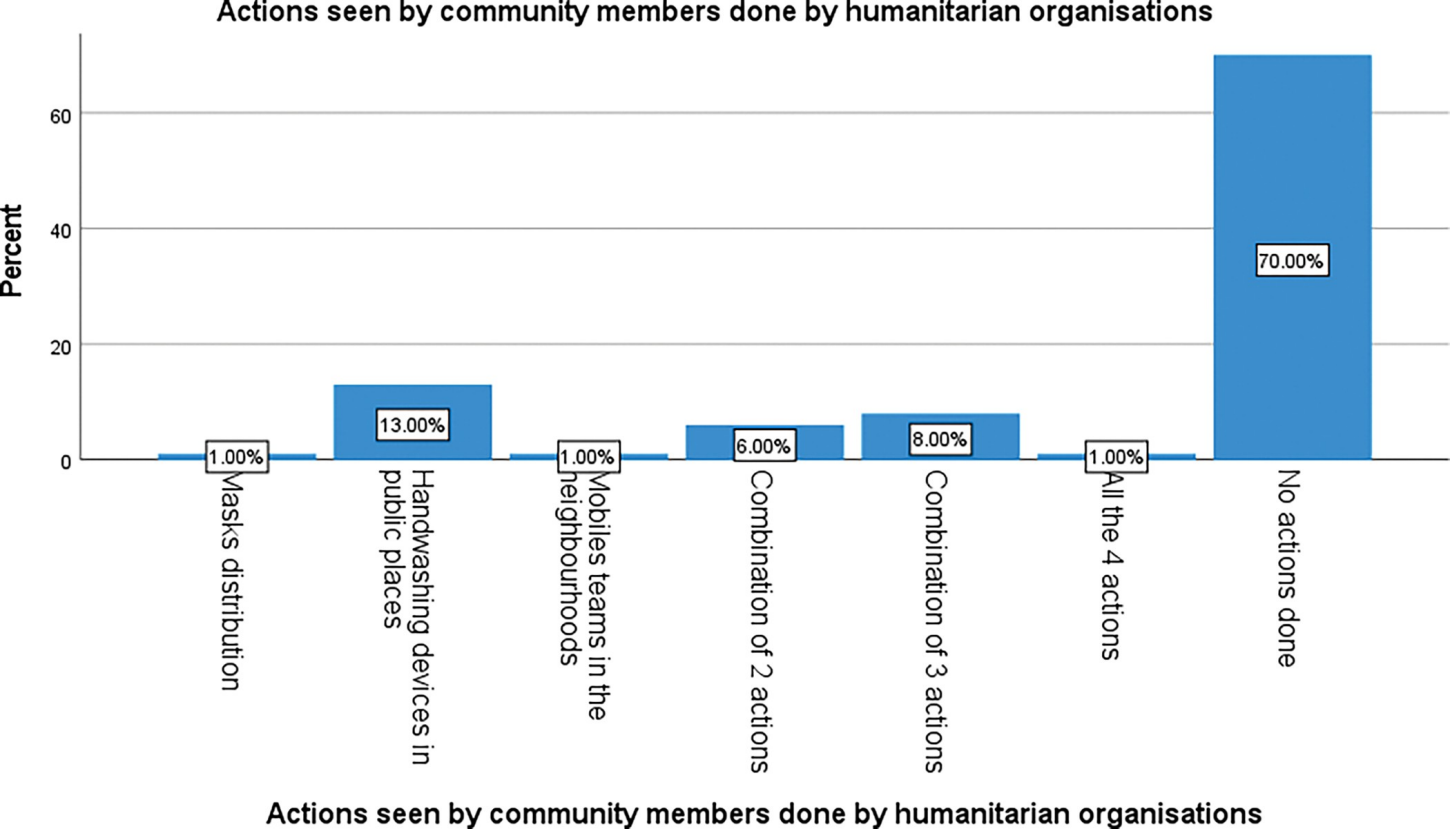
Actions seen on ground by community members done by HO.

Fig 5 shows what actions respondents said they had seen on ground according to what they expected from HO. Among our respondents 70% said they saw no actions being undertaken by HO at all, 13% saw distribution of handwashing devices, 1% saw masks being distributed, 1% saw a mobile team on ground, while 6% witnessed a combination of 2 actions, and 1% a combination of 3 interventions.

The difference observed between these categories is statistically significant, p < 0.001.

#### 3.3.4 Actions implemented versus expectations

**Fig 6:**
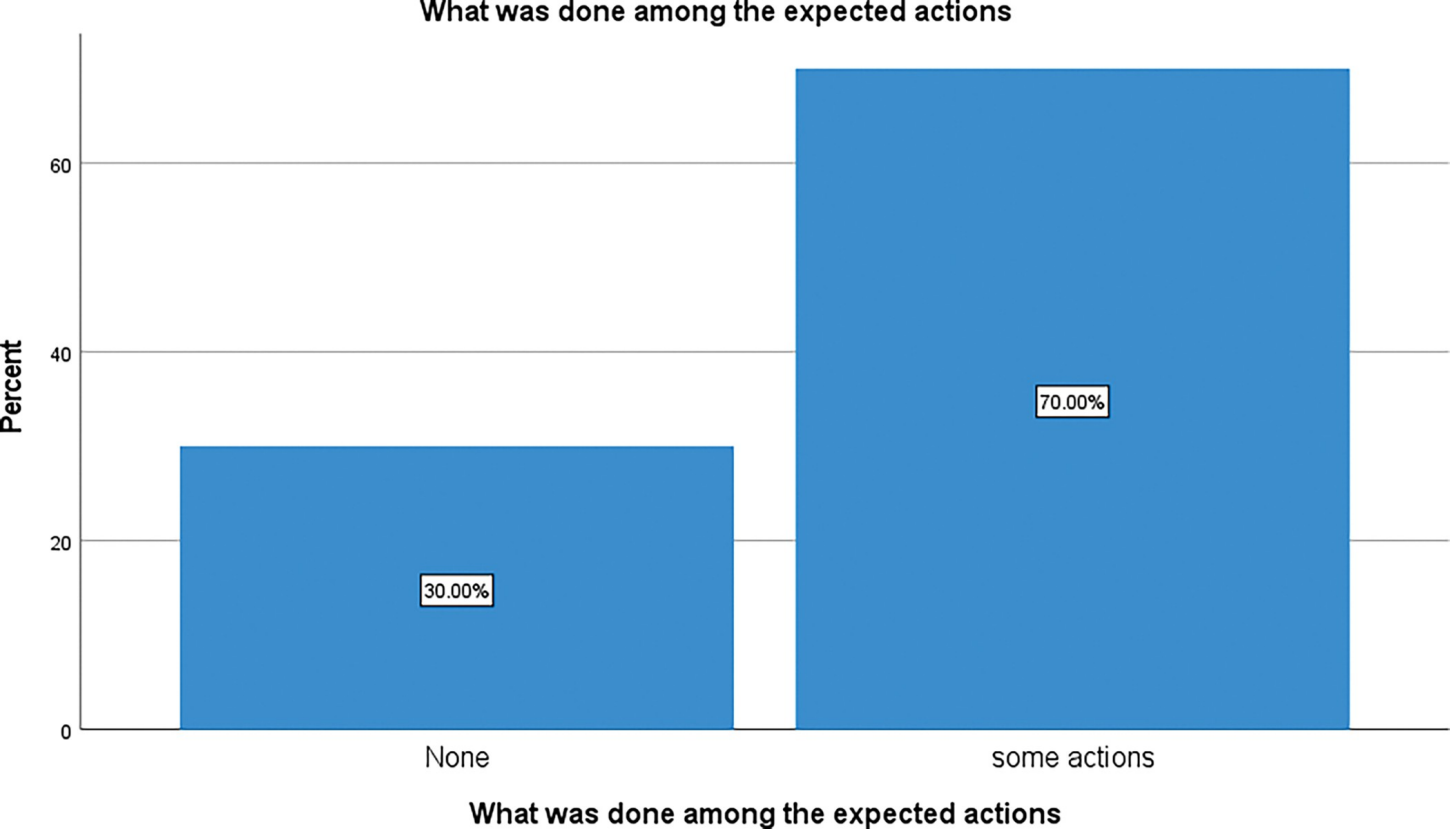
HO interventions on ground versus expectations.

When respondents were asked whether they thought the actions they expected HO to undertake were in fact done, 70% of the community members said that only some of their expectations were met whereas 30% of them thought that none of their expectations were implemented. This is summarised in Fig 6 below showing what HO implemented compared to what the community members expected.

The questionnaire was designed in a way that allow respondents to specify what are the actions they saw implemented in the community for the second choice “some actions” and this included all. Among the respondents who have chosen this option, no one has specified all. The difference observed between these categories is statistically significant, p < 0.001. Table 1 below shows the specific expectations compared to what was done and highlights a clear mismatch between what respondents expected from HO and what the HO actually implemented. The difference observed between these categories is statistically significant, p < 0.001, indicating that implemented actions did not meet community expectations.

**Table 1:** Expectations from community and reality seen on ground.

Actions	Expectations	Done
**Mask distribution**	11%	1%
**Handwashing device distribution**	32%	13%
**Mobile team on ground**	22%	1%
**Personal disinfectant distribution**	1%	-
**All the 4 actions**	33%	1%

### 3.4 Perspectives on COVID-19 vaccines

Uptake of COVID-19 vaccines was an important component of the COVID-19 response, we therefore sought to understand the sources of information on COVID-19 vaccines, whether these sources were trusted and the role HO played, if any, in trusted dissemination of information. We also asked respondents whether they were ready to accept a vaccination.

#### 3.4.1 Sources of information about COVID-19 vaccine

**Fig 7:**
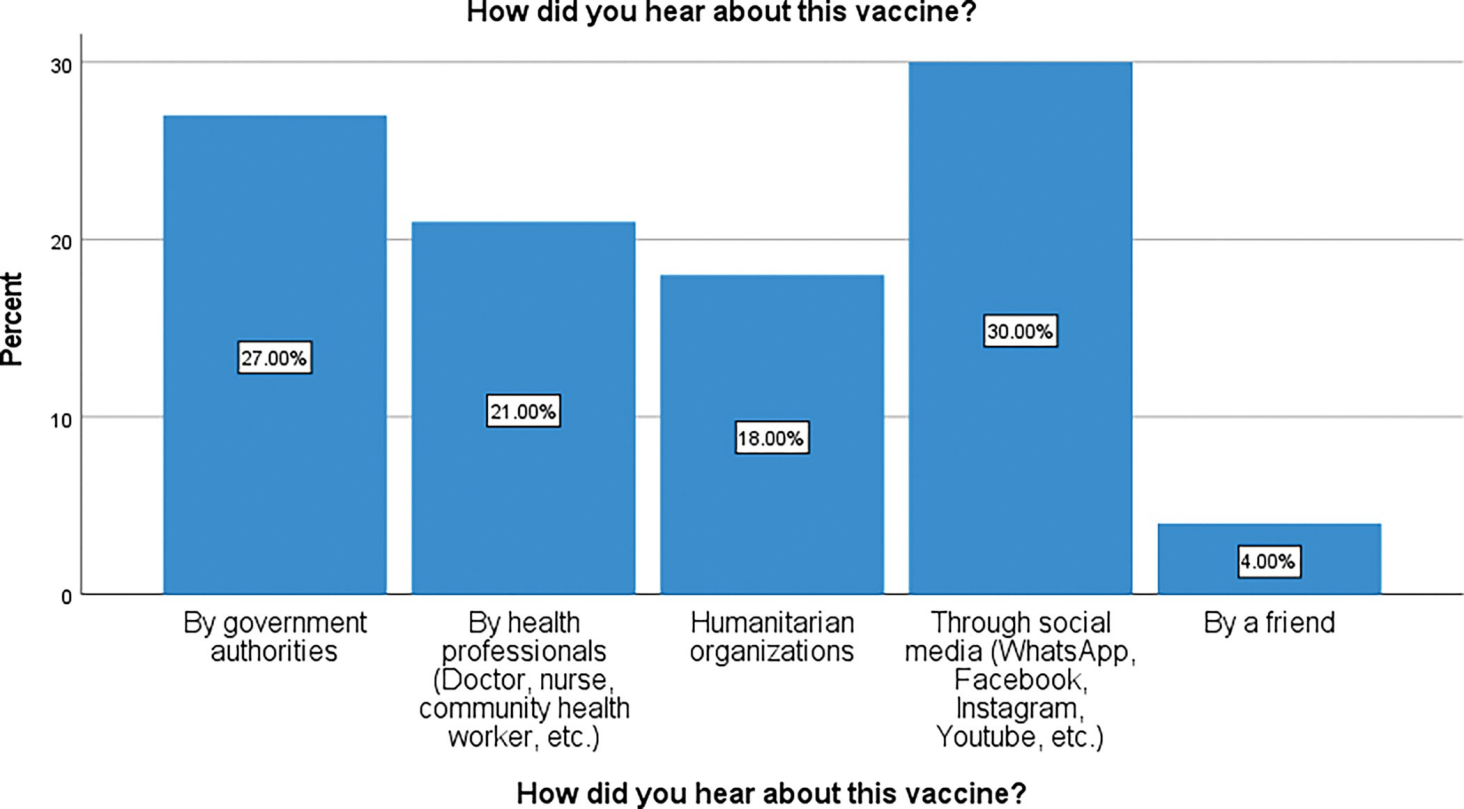
Channels of information dissemination to the community about COVID-19 vaccine.

This study found that 85% of respondents had heard about the COVID-19 vaccine and 15% had never heard about it (not shown). Fig 7 shows channels by which those respondents who had heard about the vaccine got information about it.

Most of them (30%) got their information from social media, followed by government communications (27%) and health professionals (21%). The HOs take the 4^th^ place with only 18% and some people were informed by their friends (4%). A statistically significant difference was found between these observations, p < 0.001.

#### 3.4.2 Level of trust in sources of COVID-19 vaccine information

Our study found that 78% of people in the community were ready to accept information on the importance of having a COVID-19 vaccination as a pandemic response action while 22% did not want to accept such information. It is important to understand which sources of information are most trusted.

**Fig 8:**
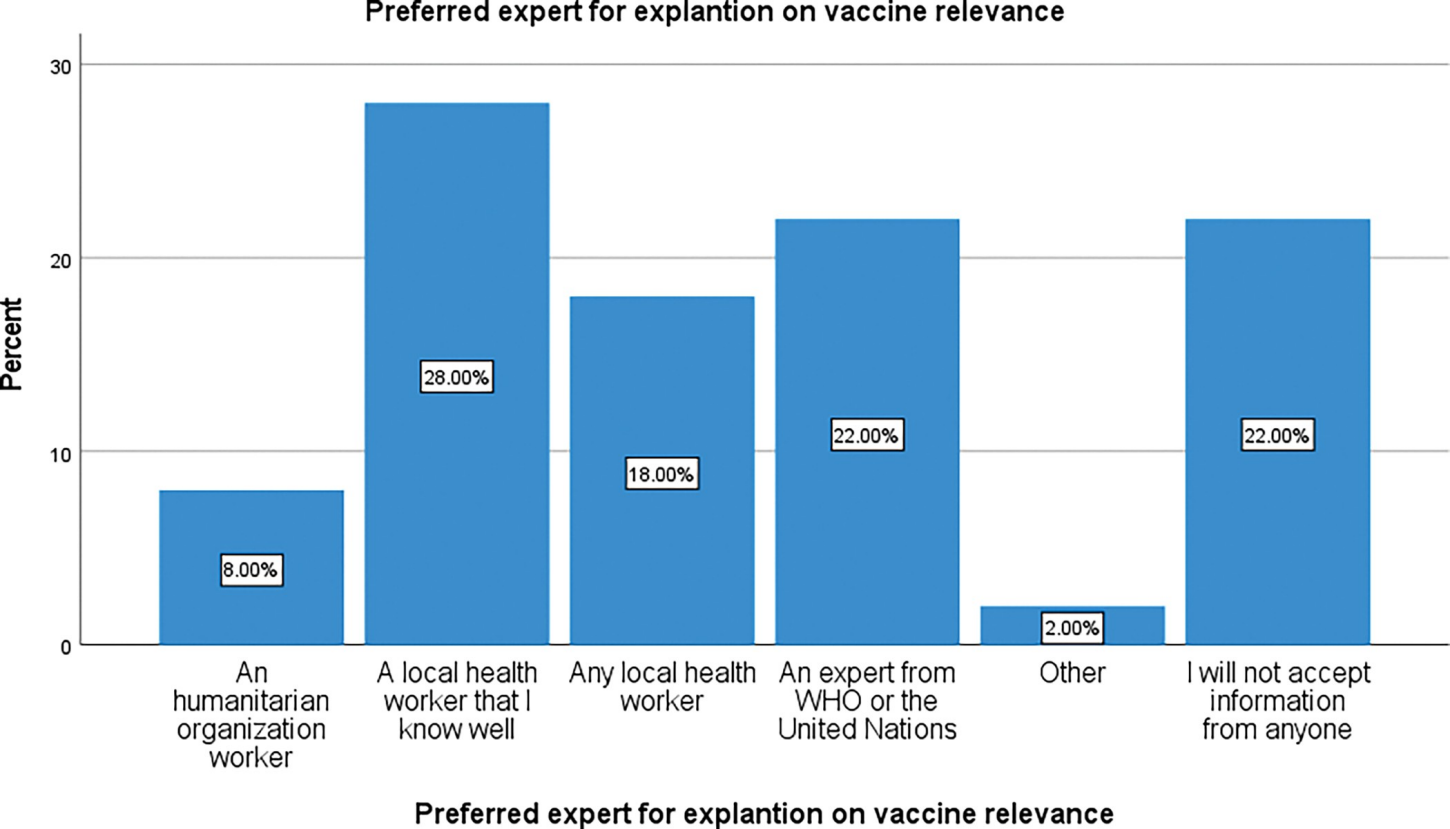
Preferred spokesperson for a talk on COVID-19 vaccine in the community.

Fig 8 shows that most people trust local health professionals that they know well (28%), followed by experts from WHO or the UN (22%), then other local health workers (18%). HO staff (who are non-governmental) are accepted by only 8% of respondents while 2% had other views. For 22% of participants, this topic was not applicable as they already said they were not willing to accept any information or explanations about COVID-19 vaccine. There a significant difference observed between these observations, p < 0.001.

#### 3.4.3 Acceptance of COVID-19 vaccine

**Fig 9:**
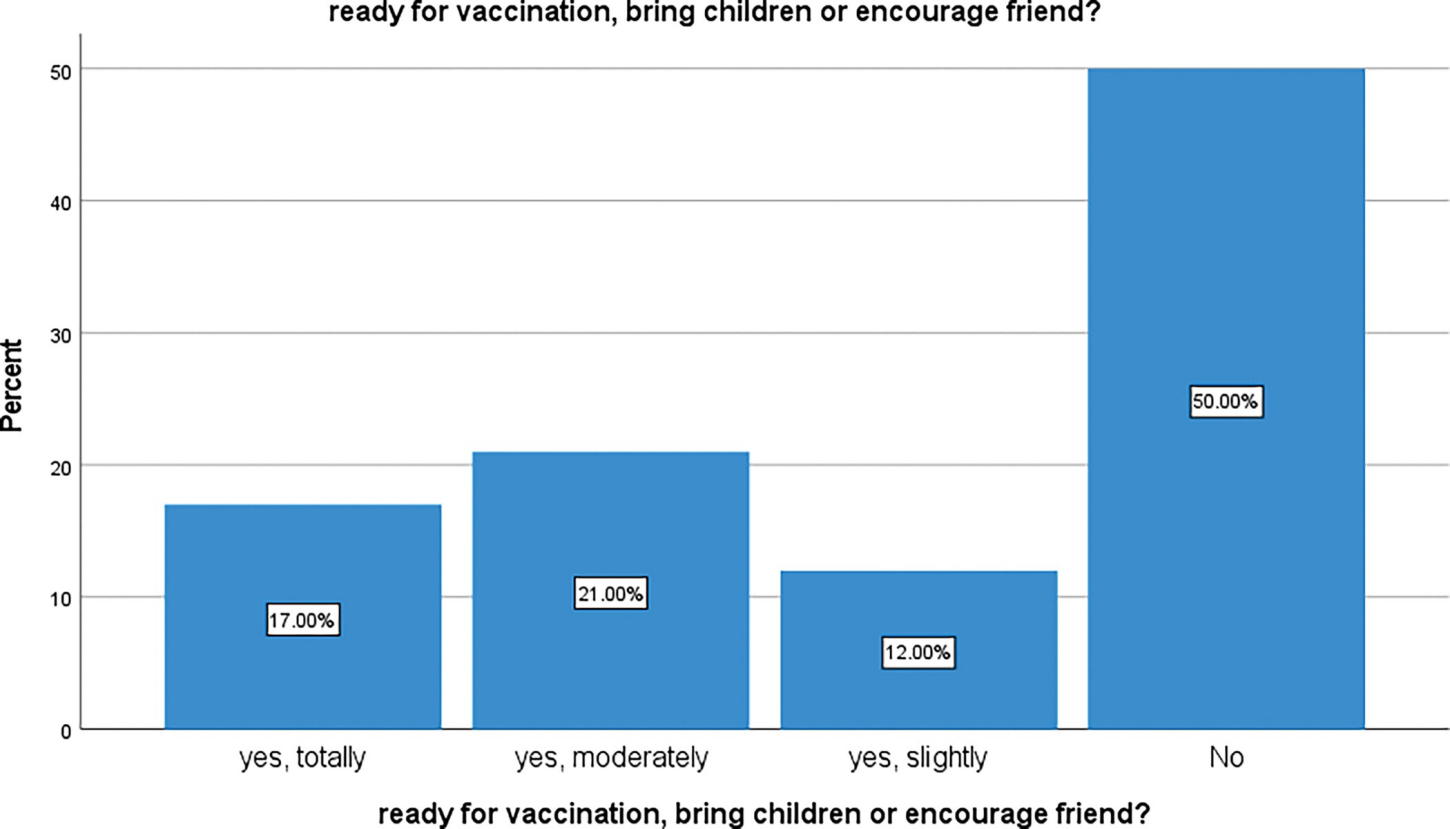
Readiness for COVID-19 vaccine uptake.

Having found that 78% people reported accepting information given to them explaining that vaccination was important, we then asked respondents whether they felt ready to actually have a vaccination (themselves, their children and encouraging their friends). Fig 9 shows results of this investigation.

We found that 50% of the participants were completely against the vaccine, 21% were moderately ready to take the vaccine, 17% were totally ready to be vaccinated, and 12% were slightly ready to take the vaccine. There is a significant difference between these observations shown by One-Sample Chi-square test, p < 0.001.

### 3.5 Respondent views by education and health profession status

Educational status is known to influence understanding of health information and health seeking behaviour [18,27,28]. There have also been reports in the literature of differences in attitude to vaccines between health professionals and non-health professions [18,27,28]. We wanted, therefore, to see whether education status or health/non-health profession status made a difference to respondents’ perceptions of HO and to their acceptance of vaccines.

#### 3.5.1 Perceptions of HO by Education level and health profession status

We explored whether respondents’ views of HO helpfulness in the COVID-19 response were shaped by their level of education and by whether or not they were health professionals. Table 2 shows the results of our investigation.

**Table 2:** Relationship between community members’ perceptions of HO presence on the ground, the education level of the participants and their healthcare/non-healthcare professional status.

		Presence of HO in the community				
		Yes, they helped us a lot	No, they were completely absent	They tried a little to help us	Total	Chi-square & p value
**Healthcare professional status**	Non-HC professionals	29 (36.7%)	32 (40.5%)	18 (22.8%)	79 (100%)	X^2^ = 0.358; p = 0.836
	HC professionals	7 (33.3%)	10 (47.6%)	4 (19%)	21 (100%)	
	**Total**	**36 (36%)**	**42 (42%)**	**22 (22%)**	**100 (100%)**	
**Educational level**	Primary school	6 (33.3%)	8 (44.4%)	4 (22.2%)	18 (100%)	X^2^ = 0.237; p = 0.994
	Secondary school	18 (35.3%)	22 (43.1%)	11 (21.6%)	51 (100%)	
	University	12 (38.7%)	12 (38.7%)	7 (22.6%)	31 (100%)	
	**Total**	**36 (36%)**	**42 (42%)**	**22 (22%)**	**100 (100%)**	

Surprisingly, the trends do not show any appreciable differences in perception either by education level or by whether respondents were healthcare professionals or not. These findings show that regardless the healthcare professional status or the level of education, the responders have the same views and understanding of the presence of HO in term of interventions to tackle the COVID-19 stress at the community level.

#### 3.5.2. Respondents’ Expectations of HO, by educational level and by healthcare professional status

Next, we explored whether respondents’ expectations of HO actions during COVID-19 response differed by educational level or health profession status. Table 3 summarises the investigation results.

**Table 3:** Community expectations from HO by categories of educational levels and healthcare professional status.

						Actions expected by community members from HO					
		Masks distribution to population	Handwashing devices in public places		Personal disinfectant distribution		Mobile team on ground	All the 4 previous actions	Others	Total	Chi-square & p value
**Healthcare professional status**	Non-HC professionals	10 (12.7%)	22 (27.8%)		1 (1.3%)		20 (25.3%)	26 (32.9%)	0 (0%)	79 (100%)	X^2^ = 8.876;p = 0.114
	HC professionals	1 (4.8%)	10 (47.6%)		0 (0%)		2 (9.5%)	7 (33.3%)	1 (4.8%)	21 (100%)	
	**Total**	**11 (11%)**	**32 (32%)**		**1 (1%)**		**22 (22%)**	**33 (33%)**	**1 (1%)**	**100 (100%)**	
**Educational level**	Primary school	4 (22.2%)	3 (16.7%)		0 (0%)		7 (38.9%)	4 (22.2%)	0 (0%)	18 (100%)	X^2^ = 12.459 p = 0.256
	Secondary school	5 (9.8%)	16 (31.4%)		1 (2%)		11 (21%)	18 (35.3%)	0 (0%)	51 (100%)	
	University	2 (6.5%)	13 (41.9%)		0 (0%)		4 (12.9%)	11 (35.5%)	1 (3.2%)	31 (100%)	
	**Total**	**11 (11%)**	**32 (32%)**		**1 (1%)**		**22 (22%)**	**33 (33%)**	**1 (1%)**	**100 (100%)**	

As for respondents’ perceptions of the helpfulness of HO presence on the ground, respondents’ expectations of what HO should do, does not differ either by educational level or by whether or not respondents were healthcare professionals.

Each of the 6 columns denotes a subset of community expectations from humanitarian organisations categories whose column proportions do not differ significantly from each other; Chi-Square test is 12.459, p = 0.256 for educational levels and Chi-Square is 8.876, p = 0.112 for healthcare professional status. This shows that the expectations were the same regardless of the educational level. Highly, moderately, and less-educated people had the same expectations from HO interventions. This is the same for healthcare professionals and non-healthcare professionals.

#### 3.5.3 Respondents’ readiness for vaccine uptake by education and healthcare professional status

Table 4 summarises the findings on whether the level of COVID-19 vaccine acceptance was different among healthcare professionals compared to non-healthcare professionals or by different levels of education.

**Table 4:** Readiness for vaccination by healthcare professional status and educational level.

		ready for vaccination, bring children or encourage friend?					
		Yes, totally	Yes, moderately	Yes, slightly	No	Total	Chi-square & p value
**Healthcare professional status**	Non-HC professionals	10 (12.7%)	16 (20.3%)	9 (11.4%)	44 (55.7%)	79 (100%)	X^2^ = 6.828; p = 0.078
	HC professionals	7 (33.3%)	5 (23.8%)	3 (14.3%)	6 (28.6%)	21 (100%)	
	**Total**	**17 (17%)**	**21 (21%)**	**12 (12%)**	**50 (50%)**	**100 (100%)**	
**Educational level**	Primary school	4 (22.2%)	5 (27.8%)	1 (5.6%)	8 (44.4%)	18 (100%)	X^2^ = 8.516; p = 0.203
	Secondary school	4 (7.8%)	12 (23.5%)	8 (15.7%)	27 (52.9%)	51 (100%)	
	University	9 (29%)	4 (12.9%)	3 (9.7%)	15 (48.4%)	31 (100%)	
	**Total**	**17 (17%)**	**21 (21%)**	**12 (12%)**	**50 (50%)**	**100 (100%)**	

Each column denotes a subset of readiness for vaccination categories whose column proportions do not differ significantly from each other, Chi-square is 6.828, p = 0.078 for healthcare professional status and Chi-square is 8.516, p = 0.203 for educational level.

This table shows that 55.7% non-healthcare professionals were not at all ready to be vaccinated, to bring their children or to recommend friends for vaccination. Among healthcare professionals, while more than half were totally or moderately willing to be vaccinated, 28.6% were completely against the vaccine. Regarding the educational level, among respondents who reached the primary school level, 22.2% were ready for vaccination whereas 44.4% were not. Among those who reached secondary school, 7.8% were fully ready for vaccination whereas 52.9% were not. In addition, among those who reached the university level, 29% were fully ready to be vaccination while 48.4% were not ready for it. The Chi-Square test, as seen in the table, shows no significant difference between these observations. This means, not either the educational level or the healthcare professional status have influenced the willingness of respondents to be vaccinated or not.

## 4. Discussion

As for many other communities worldwide, COVID-19 created fear and frustration among the population in Karisimbi Health Zone in Goma. At our community-level, 87% of participants reported being mentally affected by the presence of the disease in the community (feeling afraid) while only 12% remained unaffected (and 1% were not sure). The One-Sample p value test has shown a statistically significant difference between these categories, p < 0.0001. Our results are in line with many other studies conducted in high income countries as explained by Sampogna and colleagues in their editorial [29]. The editorial shows that all over the world, COVID-19 impacted people mentally by creating fear and frustration [29]. This was exacerbated by the forced lockdown without clear preparation for coping mechanisms and was source of untoward burden for populations and health systems.

The need for support in the COVID-19 response at community level is clear and there were a large number of international humanitarian non-governmental organisations in Goma who attempted to respond. While a lot has been written about the response of international humanitarian organisations (HO) to pandemic outbreaks [24,25,30], few studies have looked at how their efforts were perceived by the intended beneficiaries. Our findings show that expectations among community members were high with respondents saying they expected to see HO engaging in distributing handwashing devices, mobile support teams, and other activities. Yet in terms of actual presence of HO on the ground, community members were critical, with two thirds of our respondents saying they thought HO were completely absent (42%) or only a “little” in evidence (22%). Nevertheless, just over a third of respondents did see HO actions and appreciated them. Our findings on how well respondents’ expected actions matched observed actions also show a huge gap with almost no expected actions being seen on the ground except some distribution of handwashing devices and this was only observed by 13% respondents (compared to more than two thirds who expected HO to do this). While there was no difference in reported expectations and observations by respondents’ educational level or by health profession status, it is interesting to note the significant number of healthcare workers saying that HO actions on ground were not sufficient to support communities to cope with COVID-19 in Karisimbi (48% thought they were absent and 19% that they helped only a little).

There is clearly a need to create better partnerships between HO and their beneficiary communities to ensure that HO actions adequately address the expectations and needs of local populations, including local health workers. A similar study conducted by Mackworth-Young and colleagues in Zimbabwe in April 2020, also found that the COVID-19 response was not adapted to local community need and failed to meet health workers’ expectations of support, in particular the provision of personal protective equipment [16]. The Mackworth-Young study also showed that most people were not adherent to preventive measures due to lack of basic supplies of water and food. This is something that HO might be expected to provide, but was not mentioned by respondents in our study.

Other studies have noted the need to build trusted partnerships through involving local community members in pandemic and other crisis response [31–37]. These studies also demonstrated that community members can bring new ideas and become effective first responders when they are involved in every step of the response [31–37]. Indeed, the West African and DRC Ebola epidemic outbreaks have clearly shown that unless international responders are able to build trusted relationships with communities, then pandemic response efforts will be ineffective or even undermined [24,25,37]. Trust is therefore critically important for ensuring that information on a disease and important interventions like vaccines, is believed and acted upon. Our findings on people’s sources of information on COVID-19 vaccines, and their trust in these sources again show gaps and a disappointingly small contribution from HO.

Our findings show that about one third of people in Karisimbi got their information from social media, while almost half got it from official government risk communication (27%) or health professionals (21%). The role of HO was small at 18%. It is difficult to know the primary source of the information that is disseminated through social media as the HO and/or government may also be using social media. One limitation of our study is that we did not explore the social media used by politicians such as Twitter. However, most people at the grassroots level in Karisimbi do not use Twitter for communication. From common experience, the communication sent through Twitter has to be picked by a person and resent through WhatsApp or Facebook to reach the wider population at the grassroot level. Indeed, most participants in our study specified using their own friends chats or group networks on social media. Our findings on how trusted these sources were are important. Almost half of respondents said their preferred sources were local health professionals, especially if they knew them well. Another fifth said they would trust international experts (WHO, UN) but only 8% mentioned HO staff as a preferred source of information. Studies have shown that communities respond better when the responders are trusted [24,25,30,38]. Other studies have shown that using local well known community opinion leaders and local existing community structures improve the response to health shocks [30,31,39].

Our study shows quite a high level of vaccine hesitancy: half of respondents said they were not willing to take the vaccine and another 12% said they were only slightly willing. The fact that many respondents did not receive vaccine information from their preferred, or trusted, source may help explain this, but another important explanation is the views of local health professionals themselves. As noted, most people in our study community wanted–and many received–information from local healthcare professionals. Therefore, these healthcare professionals can play an important role in convincing people to take the COVID-19 vaccine. However, in our bi-variate analysis it was clear that almost third of local healthcare professionals are still not willing (or only slightly willing) to be vaccinated themselves and would not recommend the vaccine to other people. Clearly, much effort is still needed to engage healthcare professionals who could be important spokespersons to raise awareness of the benefits of COVID-19 vaccine.

Other studies in DRC and elsewhere have found similar results. In April 2020, near the beginning of the pandemic, a study conducted among healthcare workers in DRC, considering that they are the most at risk of exposure for COVID-19, showed that only 27.7% were willing to be vaccinated at that time [27]. The same year (2020), Ditekemena and colleagues also found that the willingness to be vaccinated was very low among healthcare workers as well as non-healthcare workers in DRC [18]. Our study, conducted in the late stages of the pandemic in early 2022, shows some improvement with 57% healthcare workers willing or moderately willing to be vaccinated. Another study conducted mid-pandemic, in May 2021 in Ethiopia, also showed higher proportions (64%) of community health workers who were ready to be vaccinated while 36% were not willing to take the vaccine because of poor perceptions of COVID-19 vaccine, some thinking that the vaccine can worsen pre-existing medical conditions and others have ideas that the vaccine can cause COVID-19 infection (28).

## 5. Conclusion

This study is among the first to ask community beneficiaries about their views and expectations of international humanitarian organisations. It has identified some important mis-matches between community expectations and HO actions which must be addressed in future outbreaks.

First, community members had big expectations of HO in terms of practice support to tackle the pandemic (including providing handwashing devices and mobile support teams), yet the vast majority of respondents reported seeing little or no such actions. This can create resentment against HO and it is critically important that they rapidly engage with communities at the start of any outbreak to understand their needs and concerns and develop strategies to directly respond to these.

Second, HO played a very limited role in dissemination of information about COVID-19 and were not trusted messengers. Our findings showed that most peoples’ preferred source of information about COVID-19, specifically vaccines, was local healthcare workers–particularly those who were known well and therefore trusted. Yet, many people did not receive information from these trusted sources and consequently we saw high levels of vaccine hesitancy including among healthcare workers themselves. HO (and national responders) should therefore map trusted spokespersons (including healthcare workers) in targeted communities and involve them in the planning and implementation of interventions as essential steps in the response. Among our respondents, social media played a large role in information sharing. Further research is needed to understand the role that social media (particularly Facebook and WhatsApp which were most frequently used) could play in sharing messages from trusted sources, including official government communications. Collectively, these actions could help create a positive attitude towards COVID-19 vaccine and similar interventions in future outbreaks.

## Supporting information

S1 Checklist PLOS questionnaire. (DOCX)
